# Digital Phenotyping for Detecting Depression Severity in a Large Payor-Provider System: Retrospective Study of Speech and Language Model Performance

**DOI:** 10.2196/69149

**Published:** 2025-06-19

**Authors:** Bradley Karlin, Doug Henry, Ryan Anderson, Salvatore Cieri, Michael Aratow, Elizabeth Shriberg, Michelle Hoy

**Affiliations:** 1 Highmark Health, 120 Fifth Avenue, Fifth Avenue Place, Pittsburgh, PA, 15222-3099, United States, 1 (412) 544-7000; 2 Johns Hopkins Bloomberg School of Public Health, Baltimore, MD, United States; 3 Ellipsis Health, 548 Market Street, PMB 49051, San Francisco, CA, 94104-5401, United States, 1 800-410-5383

**Keywords:** depression, vocal biomarkers, artificial intelligence, behavioral health, machine learning, health care case management, mobile phone

## Abstract

**Background:**

There is considerable need to improve and increase the detection and measurement of depression. The use of speech as a digital biomarker of depression represents a considerable opportunity for transforming and accelerating depression identification and treatment; however, research to date has primarily consisted of small-sample feasibility or pilot studies incorporating highly controlled applications and settings. There has been limited examination of the technology in real-world use contexts.

**Objective:**

This study evaluated the performance of a machine learning (ML) model examining both semantic and acoustic properties of speech in predicting depression across more than 2000 real-world interactions between health plan members and case managers.

**Methods:**

A total of 2086 recordings of case management calls with verbally administered Patient Health Questionnaire—9 questions (PHQ-9) surveys were analyzed using the ML model after the portions of the recordings with the PHQ-9 survey were manually redacted. The recordings were divided into a Development Set (Dev Set) (n=1336) and a Blind Set (n=671), and Patient Health Questionnaire—8 questions (PHQ-8) scores were provided for the Dev Set for ML model refinement while PHQ-8 scores from the Blind Set were withheld until after ML model depression severity output was reported.

**Results:**

The Dev Set and the Blind Set were well matched for age (Dev Set: mean 53.7, SD 16.3 years; Blind Set: mean 51.7, SD 16.9 years), gender (Dev Set: 910/1336, 68.1% of female participants; Blind Set: 462/671, 68.9% of female participants), and depression severity (Dev Set: mean 10.5, SD 6.1 of PHQ-8 scores; Blind Set: mean 10.9, SD 6.0 of PHQ-8 scores). The concordance correlation coefficient was ρ_c_=0.57 for the test of the ML model on the Dev Set and ρ_c_=0.54 on the Blind Set, while the mean absolute error was 3.91 for the Dev Set and 4.06 for the Blind Set, demonstrating strong model performance. This performance was maintained when dividing each set into subgroups of age brackets (≤39, 40‐64, and ≥65 years), biological sex, and the 4 categories of Social Vulnerability Index (an index based on 16 social factors), with concordance correlation coefficients ranging as ρ_c_=0.44‐0.61. Performance at PHQ-8 threshold score cutoffs of 5, 10, 15, and 20, representing the depression severity categories of none, mild, moderate, moderately severe, and severe (≥20), respectively, expressed as area under the receiver operating characteristic curve values, varied between 0.79 and 0.83 in both the Dev and Blind Sets.

**Conclusions:**

Overall, the findings suggest that speech may have significant potential for detection and measurement of depression severity over a variety of ages, gender, and socioeconomic categories that may enhance treatment, improve clinical decision-making, and enable truly personalized treatment recommendations.

## Introduction

### Background

The prevalence and impact of behavioral health (BH) problems are at an all-time high. As many as 1 in 3 individuals throughout the United States have a BH condition [1]. Rates of subclinical needs are even higher, fueled in part by the psychological and social effects of the pandemic; as many as 1 in 2 individuals reports 1 or more symptoms of depression or anxiety [2]. In fact, the prevalence of depression symptoms increased more than 3-fold during COVID-19 [3]. At the same time, only 40% of those with BH conditions, and even fewer with subclinical needs, receive care of any kind, due to challenges with and delays in detection, perceived need, stigma, a paucity of providers, and other factors, and less than 15% of individuals with serious BH conditions receive minimally adequate treatment [4]. For those who do receive care, there is an average lag time of 11 years from the time of symptom onset to first treatment, during which time symptoms often worsen and other comorbidities may develop [4].

The current state of BH care and high levels of unmet need reflect a reactive and downstream approach to the identification and treatment of BH problems that has characterized the industry for decades. Effectively and efficiently meeting BH needs requires a more proactive, upstream, and personalized approach that meets individual needs earlier in their clinical trajectories with right-sized and person-fit interventions [5]. Emerging innovations in data science and technology, particularly developments in advanced data analytics and the availability of high-quality, patient-driven digital interventions, present unprecedented opportunities to transform and innovate the field of BH care and reduce enduring, high rates of unmet need.

One particularly promising innovation for advancing detection and delivery of proactive, personalized, and data-informed treatment is digital phenotyping. Digital phenotyping involves the detection of phenotypes, or behavioral signals, that may indicate or predict the presence of a BH problem. Translated by machine learning (ML) models and collected through passive data collection via smartphones, wearables, or other communication devices, these data signals may serve as clinically, and potentially preclinically, useful markers of BH problems. The potential relevance and use of digital phenotyping, which has been identified as the “next frontier” for personalized and proactive care within the field of oncology [6], have attracted particular interest and attention in the field of BH care and personalized psychiatry, with recent calls for accelerated applications to clinical practice [7-9]. In their review of research in this area, Huckvale et al [7] declared, “Many...studies appear to anticipate that digital phenotyping should play a role in routine clinical practice, for example by enhancing aspects of clinical diagnosis and treatment through earlier detection of condition onset, relapse or treatment response.”

Most of the research examining digital phenotyping for the detection of BH problems has focused on detection of depression [10]. The opportunity to engage passive and objective ML technology for better detecting depression presents particular opportunities in light of the fact that depression is undetected in approximately 50% of individuals with the condition in high-income countries, and in 80%‐90% of individuals with depression in low- and middle-income countries [11]. In addition to opportunities that automated detection of depression provides for increasing low screening rates in most clinical and community settings, ML presents significant promise for overcoming underreporting and underdetection due to stigma, lack of evaluative service access, misattribution of symptoms to physical illness or age-related factors, or underrecognition of symptoms. In addition, the use of ML for detecting depression offers significant potential for increasing *earlier* identification and intervention, enhancing clinical efficiency through more accurate triage and treatment performance monitoring, improving fidelity through the use of objective measures, providing decision support, and personalizing BH care. As Galatzer-Levy and Onnela [12] recently declared, “Ultimately, the development of clinically meaningful digital measurements and their implementation in real-world contexts will permit optimized and personalized treatments targeted to the individual’s emergent presentation and needs.”

### Prior Work

Among the most promising applications of digital phenotyping is the use of speech as a vocal biomarker of depression and other BH conditions [13]. The application of speech analysis in this context includes models for moment-by-moment analysis of the semantic patterns (“what” is said) or the acoustic properties (eg, tone, pitch, loudness, duration, articulation, transitions, and prosody) of speech, or the application of both. Increasing research has demonstrated the promise of speech analysis, including generally increasing accuracy in overall detection of depression and other conditions [7]. Despite this promise, research to date has been primarily conducted in controlled contexts and uses, and there has been very limited examination or application of this technology in real-world settings [7,14]. As Koutsouleris et al [14] recently noted, “While these innovations promise to revolutionize health care, little progress has been made toward real precision mental health applications. Implementation of these applications is often an afterthought.”

Research on the use of speech analysis for measuring BH symptoms has consisted primarily of small-sample feasibility or pilot studies with nonrepresentative samples [7,14-16]. For example, in a scoping review of speech analysis for measuring mood disorders conducted by Flanagan et al [15], approximately 80% of studies were pilot or feasibility studies with sample sizes ranging from 1 to 73 participants. Similarly, in their review, Chia and Zhang [10] reported a mean sample size of 60. Moreover, many studies have consisted of analysis of “toy” datasets or controlled proof-of-concept studies involving highly controlled designs that, while promising for establishing the potential of a technological innovation, have yielded findings that are not necessarily generalizable or have use or effectiveness for real-world use [10,17]. These designs include use of analog speech tasks (eg, responding to a singular question, reading formulated passages, and answering questions about everyday life, often referred to as “closed-form” tests) that are often not comparable with real-world clinical settings or real-life contexts.

In addition, many studies examining speech analysis in the BH context have had important methodological limitations, including frequent reporting of selected metrics, such as reporting of sensitivity without specificity, leading to the recent call for research in this area to report multiple metrics, including robust metrics, such as the concordance correlation coefficient (CCC), that are not as biased to specific context, use case, and data label distributions [13]. Many studies have also relied on binary classifications (above or below cutoff score for clinical significance) for screening tools, which limit opportunities for promoting precision and personalization in BH care. Furthermore, research on speech analysis in detecting and measuring BH symptoms has almost exclusively relied on the use of single methods of analysis (predominantly acoustic analysis). Opportunities for leveraging and combining analysis of acoustic and semantic properties of speech may yield greater accuracy and precision in detecting and predicting BH conditions.

### Goal of This Study

As mentioned previously, the application of digital phenotyping within BH care has approached a defining moment and key turning point for the field. In their review of the current state of digital phenotyping within the field of BH, Huckvale et al [7] have urged for “practical and coordinated action...to help accelerate both research and the ultimate development of real-world health applications for digital phenotyping.” In an effort to help advance real-world application of digital phenotyping for promoting earlier and automated detection and measurement of depression, this study evaluated the performance of an ML model of the semantic and acoustic properties of spoken language in predicting depression in a naturalistic context by analyzing more than 2000 interactions between health plan members and case managers. Additionally, the study sought to test model performance beyond “presence or absence” dichotomous predictions, examining classificatory accuracy at multiple levels of depression from none or minimal to severe. Furthermore, model performance was tested across age, sex, and sociodemographic factors and in BH and non-BH case management contexts. This project, which is unique in its breadth and scope, aims to assess the accuracy of speech analysis for detecting and measuring depression severity in routine clinical settings. We hypothesize that the ML models used in this study will demonstrate robust predictive accuracy across variations in age, gender, care management context, and Social Vulnerability Index (SVI).

## Methods

### Experimental Design

The current quality improvement project evaluated the performance of the combined semantic-acoustic ML speech analysis model in predicting depression severity from existing recordings of case management calls, with BH case managers who are licensed independent mental health providers. Specifically, the performance of the ML model in care management conversations between insured members and BH case managers was evaluated by retrospectively comparing the actual scores from the Patient Health Questionnaire—9 questions (PHQ-9) administered by the case managers. The predicted Patient Health Questionnaire—8 questions (PHQ-8) scores were derived from the qualities (acoustic biomarkers and semantic content) of vocal productions of the same members conversing with care managers while engaged in discussion other than the PHQ-8 administration. It was secondarily sought to examine model performance in non-BH contexts where the PHQ-9 is not routinely administered using a subsample of calls with non-BH case managers. For both BH and non-BH calls, model predictions were compared with PHQ-8 scores from an associated metadata file.

### Ethical Considerations

On each of the calls analyzed, the PHQ-9 was verbally administered. Members consented to the recording of the call for quality and training purposes. This study was designated as a quality improvement project by the institutional review board of the Allegheny Health Network and therefore exempt from ongoing institutional review board oversight. The project was also reviewed and approved by the Highmark Health Enterprise Data Governance Committee to ensure that it comported with internal data protection standards and applicable privacy, legal, and regulatory requirements, including deidentification of data. There was no compensation provided as recordings were made in the normal course of business.

### Measures

#### Depression Severity

The PHQ-9 is a widely used self-report measure of depression symptom severity. Frequency of depression symptoms are endorsed by patients using a 4-point scale, ranging from 0 (“Not at all”) to 3 (“Nearly every day”). PHQ-9 scores range from 0 to 27. Higher scores reflect greater depression severity. Scores of 0-4 are classified as “none to minimal,” 5-9 are classified as “mild,” 10-14 are classified as “moderate,” 15-19 are classified as “moderately severe,” and 20-27 are classified as “severe.” The PHQ-9 has been shown to be an internally consistent, valid, and reliable measure of depression severity [18,19]. For this study, the last item of the PHQ-9, which assesses for suicidal or self-injurious thoughts, was omitted given the use of archival data where further probing of responses was not feasible. Its inclusion requires different clinical considerations and handling in research settings. The adapted scale with item 9 removed is referred to as the PHQ-8 and has been shown to have strong psychometric characteristics, including the ability to accurately predict depression [20].

#### ML Speech Analysis Model

The semantic-acoustic model evaluated in this study has demonstrated robust results for accurate prediction of depression symptom severity and acceptable rates of error [21-23]. The proprietary ML model includes both acoustic and semantic models. The acoustic model takes as input the raw speech signal (rather than precomputed features such as pitch or energy). The production acoustic workflow is built on a pretrained open-source wav2vec2 architecture [24] and is trained on proprietary audio data. The system consists of 4 segment models, each trained with specific configurations, and 3 segment fusion models that integrate outputs from the segment networks. Predictions from the segment fusion models are weighted to generate the final acoustic score.

The semantic model (referred to also as a natural language processing model) takes as input the output of a commercial automatic speech recognition (ASR) system. The model is based on the Longformer architecture [25], designed to efficiently handle long conversational contexts using advanced mechanisms such as dilated sliding window attention. Model training involves a proprietary fine-tuning approach using depression-specific data, using high-quality proprietary transcripts paired with PHQ scores. Further refinement is conducted using conversational samples, also labeled with PHQ scores. Labeled training data come from a large corpus of proprietary spoken language datasets labeled with PHQ-8 values. Both models take advantage of publicly available data for model pretraining, including text corpora for the natural language processing model.

**Figure 1.**
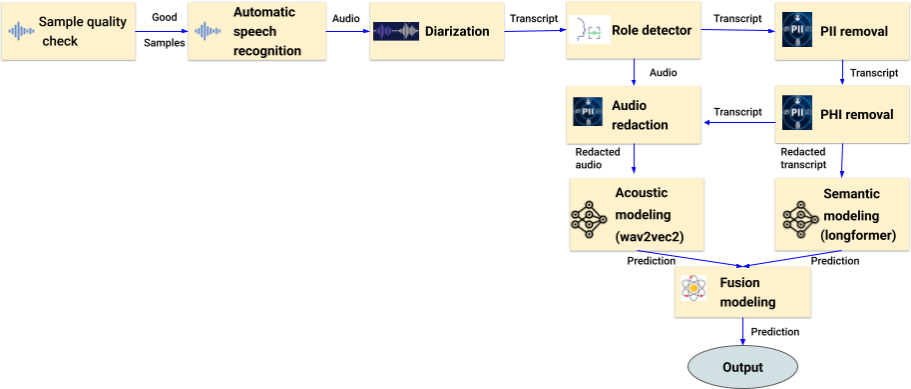
Deep learning architecture and processing pipeline. PHI: Protected health information; PII: personally identifiable information.

To generate the final depression severity prediction, the outputs of the acoustic and language model are combined using a linear weighting; the weight is optimized using the CCC metric on the Development set. Figure 1 illustrates the overall ML analysis, from data preprocessing to prediction generation.

### Identification of Case Management Calls and Metadata

A total of 2626 recordings of case management calls were included. They took place between January 2019 and January 2023. Of these calls, 2083 had full item-level data for the first 8 items of the PHQ-9, which were collected verbally during the course of calls. Calls corresponded to unique members from 44 different US states and were completed by 46 case managers. The majority of case managers completed multiple calls, and approximately one-third completed 20 or more calls. Each call recording had an associated metadata file containing member age, biological sex, zip code, whether the call was conducted by a BH case manager or non-BH (eg, medical and surgical) case manager, and PHQ-9 item-level data. Exclusion criteria for recordings included member age less than 18 years, speechmail messages, presence of any speakers beyond the case manager and the member, recordings in which the member was not present, and diarization failures (failures to correctly segment audio into single-speaker time regions). These exclusion criteria constituted 76 of the 2083 calls, leaving a total of 2007 calls for the analysis.

### Partitioning the Data

The evaluation was conducted in 2 phases. To establish datasets for both phases of the project, the 2007 recordings and their metadata were partitioned by randomly assigning them to a development dataset (Dev Set) consisting of approximately two-thirds of the total available calls (n=1336) and a test dataset (Blind Set) consisting of approximately one-third of the total available calls (n=671). There was no speaker overlap across these datasets. The partitions were constructed to ensure reasonably equal representation of the metadata, including the distribution of PHQ-9 severity (none, mild, moderate, moderately severe, and severe). The Dev Set and Blind Set were securely delivered via secure file transfer protocol to Ellipsis Health, which performed all further data processing and analyses of the calls and metadata. The Blind Set was held back until phase 1 was completed.

The 2 datasets (Dev and Blind) were well matched; however, there were data curation errors such as inclusion of voicemails, conversations in a different language (mostly involving a language interpreter), and minors (younger than 18 years). Subsequent to the delivery of each dataset, 76 recordings, 44 from the Dev Set and 32 from the Blind Set, were found to meet exclusionary criteria through review of metadata (ie, age) and through diarization tool flags indicating a single speaker or more than 2 speakers. These 76 recordings were removed from the analyses. However, because the audio tracks were not reviewed by the annotators, other recordings meeting exclusionary criteria were included in the Dev and Blind Sets. An analysis was conducted, and it is estimated that inclusion errors constituted 3% of the total recordings analyzed.

### Data Preprocessing

Upon receiving call recordings, Ellipsis Health performed diarization, ASR, and redaction of personally identifiable information using Amazon Web Services Amazon Transcribe [26]. Redaction of protected health information was performed using Amazon Comprehend Medical [27]. Speaker role detection and time stamp generation on turns in conversation (ie, transition from member to case manager and vice versa) were performed using proprietary algorithms from Ellipsis Health. The redacted output of the ASR process, which included transcripts of both the member and the case manager with PHQ-9 content removed, was used by the semantic model. Meanwhile, the diarized, redacted audio containing only the member’s speech, with PHQ-9 content masked, was used by the acoustic model. The outputs of the semantic and acoustic models were used (after weighting) to arrive at the fused output, using the Dev dataset.

### Manual Annotation

To remove the verbally administered PHQ-9 from the calls, a manual annotation process was performed to identify the regions in the transcripts where the PHQ-9 was administered. Ellipsis Health used a team of professionals separate from the team conducting tests of the ML model (ie, ML team) to perform manual annotation, which consisted of annotators being presented with the case manager portion of the transcript from each call and having them identify the regions that contained the PHQ questions. These annotated regions of audio samples were masked in white noise for the acoustic model analysis, and the corresponding text was removed from the transcripts for the semantic model analysis.

### Model Refinement on the Dev Set

In phase 1, the semantic-acoustic model was applied to the Dev Set (n=1336), and hyperparameters (eg, learning rate in optimization algorithms, number of hidden layers, and number of iterations in training a neural network) were optimized to minimize the CCC [28].

### Tests on the Blind Set

Phase 2 of the project was conducted to evaluate the performance of the semantic-acoustic ML model established in phase 1. The Blind Set used in phase 2 was provided to Ellipsis Health without any accompanying PHQ-9 scores to ensure a blinded test of the model. Other than absence of PHQ-9 scores, the provided metadata categories were the same categories as provided for the Dev Set. ASR was conducted, followed by personally identifiable information and protected health information redaction of both the audio and transcript, using the same process as for the Dev Set. Manual annotation of the recordings was performed as in phase 1 and the verbally administered PHQ-9 was masked in the audio file and removed from the call transcript. The ML team then conducted tests of the ML model to predict depression severity scores for the Blind Set and across the metadata subgroups of the Blind Set. Recorded PHQ-9 scores from the original calls in the Blind Set were subsequently provided to the ML team, and PHQ-8 scores were then derived from these PHQ-9 scores and then compared with ML model predictions of the PHQ-8 scores to complete the test of model accuracy.

In light of the fact that overreporting and underreporting are well-known phenomena of surveys, including on sensitive measures such as the PHQ-9 [28-30], a preliminary exploration of the possible presence of such when responding to the PHQ-9 was conducted by examining for sizeable discrepancies between PHQ-8 labels and predicted depression scores. Overreporting and underreporting were defined as a difference of ≥2 categories of classification between the model prediction and the PHQ-8 score, as this would likely cause a significant change in a care pathway for a patient, and this condition was found in 42 of the 2007 total recordings. Five licensed therapists were recruited to listen and rate the member for severity of depression symptoms (none, mild, moderate, and severe). They were assigned recordings such that 1 therapist listened to each of the 42 recordings, but in 25 of those calls at least 2 therapists provided an additional assessment. The therapists were blinded to all information, including the PHQ score and section of the recording where the survey was administered, the model predictions, and demographic information. A PHQ-8 score predicted by ML model was defined as agreeing with a mental health provider assessment if their assessment was the same or within 1 severity category difference.

### Metrics

The ML model results included CCC, mean absolute error (MAE), area under the receiver operating characteristic curve (AUROC), and sensitivity and specificity at the point of equal error rate (EER) for the Dev Set and the Blind Set. All classification analyses were conducted with the PHQ-8 as the criterion or observed score. Predicted scores from the ML regression models were binned according to the following PHQ-8 depression severity classifications: none or minimal (0‐4), mild (5-9), moderate (10-14), moderately severe (15-19), and severe (20-24). Next, ROC analyses were conducted, comparing predicted to observed scores across 5 binary classifications at the 4 PHQ-8 cutoffs (5, 10, 15, and 20): 0-4 versus 5-24, 0-9 versus 10-24, 0-14 versus 15-24, and 0-19 versus 20-24. AUROC and sensitivity and specificity at the EER were calculated at each cutoff and reported for the ML model.

## Results

### Comparison of Member-Level Metadata

Data demographic distributions for members were comparable across both datasets. Ages ranged from 18-98 years in the Dev Set and 18-92 years in the Blind Set (Table 1). Approximately two-thirds of members across the Dev Set (910/1336, 68.1% of participants) and Blind Set (462/671, 68.9% of participants) were female. Member zip code was used to establish the SVI [31], which is based on 16 social factors, including socioeconomic status (eg, below poverty and unemployed), household characteristics (eg, single parent and aged 65 years or older), and housing type or transportation (eg, crowding and no vehicle). In each dataset, members were predominantly in the low-moderate range for social vulnerability and the majority of calls were BH case management calls (Table 1).

**Table 1.** Distribution of metadata for the Dev and Blind Sets.

Metadata	Dev Set (n=1336)	Blind Set (n=671)
Age (years), mean (SD), range	53.7 (16.3), 18‐98	51.7 (16.9), 18‐92
Age (years), n (%)		
≤39	296 (22.2)	183 (27.3)
40‐64	704 (52.7)	344 (51.3)
≥65	336 (25.1)	144 (21.4)
Biological sex, n (%)		
Female	910 (68.1)	462 (68.9)
Male	426 (31.9)	207 (30.8)
Undefined	0 (0)	2 (0.3)
SVI^a^, n (%)		
1	279 (20.9)	150 (15.6)
2	617 (46.2)	306 (45.6)
3	335 (25.1)	162 (24.1)
4	102 (7.6)	51 (7.6)
Missing	3 (2.2)	2 (0.3)
Type of CM^b^, n (%)		
BH^c^	1087 (81.4)	561 (83.6)
Non-BH	249 (18.6)	110 (16.4)
PHQ-8^d^, mean (SD), range	10.5 (6.1), 0‐24	10.9 (6.0), 0‐24
PHQ-8^e^, n (%)		
None or minimal	249 (18.6)	113 (16.8)
Mild	384 (28.7)	179 (26.7)
Moderate	328 (25.6)	174 (25.9)
Moderately severe	263 (19.7)	141 (21.0)
Severe	112 (8.4)	64 (9.5)

a SVI: Social Vulnerability Index (1 = least vulnerable, 4 = most vulnerable).

b CM: case management.

c BH: behavioral health.

d PHQ-8: Patient Health Questionnaire—8 questions.

e None or minimal=0‐4, mild=5‐9, moderate=10‐14, moderately severe=15‐19, and severe=20‐24. Percentages may not add up to 100% due to rounding.

### Regression Results for Overall Tests of the ML Model

Results for the test of the ML model on the Dev Set (n=1336) produced a CCC of ρ_c_=0.57, which is superior to results expected by chance (ρ_c_=0.10-0.20). CCC showed minimal decrease in the test of the ML model on the Blind Set (ρ_c_=0.54; n=671). Furthermore, MAE values for the ML model tests across datasets were 3.91 and 4.06 for the Dev Set and Blind Set, respectively. These values for MAE are equivalent to less than the score range (5 points) of a single PHQ-8 severity classification.

### Classification Results for Overall Tests of the ML Model

The AUROC at PHQ-8 cutoff of 10 (ie, “moderate” depression, the traditional cutoff for the majority of clinical care pathways [31,32]) was consistent for the ML model as applied to the Dev Set (0.83) and Blind Set (0.81), which are identical to the respective mean AUROC values over the different cutoff points (Table 2, top panel). In particular, results for the ML model (AUROC=0.81) on the Blind Set indicate the robustness of the model in its ability to identify individuals with PHQ-8 scores above 10 using novel call data (ie, data without PHQ-8 labels and not previously used for model refinement).

**Table 2.** Regression and classification results for overall tests of the machine learning model.

Statistic	Dev Set (n=1336)	Blind Set (n=671)
CCC^a^	0.57	0.54
MAE^b^	3.91	4.06
AUROC^c^		
Mean^d^ across cutoffs^e^	0.83	0.81
Cutoff 5	0.81	0.85
Cutoff 10	0.83	0.81
Cutoff 15	0.83	0.79
Cutoff 20	0.83	0.79
Sens=Spec^f^		
Mean across cutoffs	0.74	0.73
Cutoff 5	0.73	0.76
Cutoff 10	0.75	0.72
Cutoff 15	0.74	0.72
Cutoff 20	0.76	0.72

a CCC: concordance correlation coefficient.

b MAE: mean absolute error.

c AUROC: area under the receiver operating characteristic curve.

d Mean across 4 cutoffs (5, 10, 15, and 20).

e Cutoff numbers were chosen as these are the points where the depression severity category boundaries occur.

f Value of both sensitivity and specificity at point of equal error.

AUROC values across the 4 cutoff thresholds and across both datasets ranged from 0.79 to 0.85 (Table 2). The lowest AUROCs were observed for the ML model (AUROC=0.79) on the Blind Set at PHQ-8 cutoffs of 15 and 20 (“moderately severe” and “severe” depression). Of note, the size of the subsample of members in the Blind Set with scores ≥20 was only 64 members and may have contributed to the lower AUROC values for this classification. See Table 1 for information on sample sizes across classifications for all 3 datasets.

As shown in Table 2, the mean sensitivity and specificity at the point of equal error across the 4 classifications was stable for ML model performance. Across the 4 PHQ-8 cutoff scores, sensitivity and specificity values ranged from 0.72 at a cutoff of 10, 15, and 20 for the ML model test on the Blind Set to 0.76 for the ML model test on the Dev Set at a cutoff of 20 and on the Blind Set at a cutoff of 5. As observed with AUROC, values at the lower end of the range for sensitivity and specificity may have been affected by smaller subsample sizes (eg, Blind Set with PHQ-8 ≥20).

### Model Performance Across Metadata Subgroups

ML model performance was evaluated across metadata subgroups based on age in years (18‐39, 40‐64, and ≥65), sex (male and female), and BH case management versus non-BH case management and across the 4 SVI levels (1=least vulnerable and 4=most vulnerable).

The ML model performance between the 2 datasets (Dev Set and Blind Set) within their subgroups (age [Table 3], sex [Table 4], type of case management call [Table 5], and SVI [Table 6]) reveals both consistent and relatively similar AUROC cutoff at 10 and EER values with AUROC cutoff at 10 ranging from 0.81 to 0.83 and sensitivity and specificity at point of equal error ranging from 0.73 to 0.75, implying good model stability and robustness. See Figures S1-S12 in Multimedia Appendix 1 for ROC curves (overall and per subgroup on Blind Set). CCC ranged from 0.44 to 0.61, with the lowest (0.44) occurring in the most highly socially vulnerable group in the Blind Set and the highest (0.61) occurring in both the least socially vulnerable group of the Dev Set and the ≥65 years age group in the Blind Set. In most cases, the lower CCC values occurred where sample sizes were approximately 100 or fewer individuals, and our previous work [33] suggests a minimum count of approximately 200 individuals for robust estimates of prediction performance. MAE values ranged from 3.62 in the ≥65 years age group of the Blind Set to 4.57 in the non-BH group of the Blind Set. The 2 highest MAE values were associated with subgroups with sample sizes of approximately 100 or fewer participants, comparable with results for the CCC.

**Table 3.** Regression and classification metrics for model tests by the subgroup age.

	Dev Set (n=1336)			Blind Set (n=671)		
	Aged ≤39 years (n=296)	Aged 40‐64 years (n=704)	Aged ≥65 years (n=336)	Aged ≤39 years (n=183)	Aged 40‐64 years (n=344)	Aged ≥65 years (n=144)
CCC^a^	0.58	0.55	0.58	0.57	0.47	0.61
MAE^b^	3.91	4.00	3.77	3.93	4.32	3.62
AUROC^c^ cutoff^d^ 10	0.83	0.83	0.83	0.81	0.81	0.81
Sensitivity and specificity^e^ cutoff 10	0.76	0.75	0.75	0.72	0.72	0.72

a CCC: concordance correlation coefficient.

b MAE: mean absolute error.

c AUROC: area under the receiver operating characteristic curve.

d Cutoff thresholds correspond to the boundaries of clinical depression severity classes.

e Value of both sensitivity and specificity at point of equal error.

**Table 4.** Regression and classification metrics for model tests by the subgroup sex.

	Dev Set (n=1336)		Blind Set (n=671)	
	Female (n=910)	Male (n=426)	Female (n=462)	Male (n=207)
CCC^a^	0.56	0.58	0.53	0.54
MAE^b^	3.86	4.04	3.95	4.29
AUROC^c^ cutoff^d^ 10	0.83	0.83	0.81	0.81
Sensitivity and specificity^e^ cutoff 10	0.75	0.75	0.72	0.72

a CCC: concordance correlation coefficient.

b MAE: mean absolute error.

c AUROC: area under the receiver operating characteristic curve.

d Cutoff thresholds correspond to the boundaries of clinical depression severity classes.

e Value of both sensitivity and specificity at point of equal error.

**Table 5.** Regression and classification metrics for model tests by the subgroup type of case management.

	Dev Set (n=1336)		Blind Set (n=671)	
	BH^a^ (n=1087)	Non-BH (n=249)	BH (n=561)	Non-BH (n=110)
CCC^b^	0.57	0.58	0.55	0.46
MAE^c^	3.92	3.86	4.00	4.40
AUROC^d^ cutoff^e^ 10	0.83	0.83	0.81	0.81
Sensitivity and specificity^f^ cutoff 10	0.75	0.75	0.72	0.72

a BH: behavioral health.

b CCC: concordance correlation coefficient.

c MAE: mean absolute error.

d AUROC: area under the receiver operating characteristic curve.

e Cutoff thresholds correspond to the boundaries of clinical depression severity classes.

f Value of both sensitivity and specificity at point of equal error.

**Table 6.** Regression and classification metrics for model tests by the subgroup Social Vulnerability Index (SVI).

	Dev Set (n=1336)				Blind Set (n=671)			
	SVI^a^=1 (n=279)	SVI=2 (n=617)	SVI=3 (n=335)	SVI=4 (n=102)	SVI=1 (1n=50)	SVI=2 (n=306)	SVI=3 (n=162)	SVI=4 (n=51)
CCC^b^	0.61	0.57	0.57	0.46	0.61	0.54	0.47	0.44
MAE^c^	3.76	3.90	3.98	4.24	3.83	4.04	4.18	4.57
AUROC^d^ cutoff^e^ 10	0.83	0.83	0.83	0.83	0.81	0.81	0.81	0.81
Sensitivity and specificity^f^ cutoff 10	0.75	0.75	0.75	0.75	0.72	0.72	0.72	0.72

a SVI: Social Vulnerability Index (1=least vulnerable, 4=most vulnerable).

b CCC: concordance correlation coefficient.

c MAE: mean absolute error.

d AUROC: area under the receiver operating characteristic curve.

e Cutoff thresholds correspond to the boundaries of clinical depression severity classes.

f Value of both sensitivity and specificity at point of equal error.

Finally, within the Dev Set, 3.1% (42/1336) of recordings showed sizable discrepancies (divergence equivalent to 2 or more PHQ-8 categories) between administered PHQ-8 and ML-predicted depression score that could imply PHQ-8 response underreporting (actual depression score much lower than predicted depression score) or overreporting (actual depression score much higher than predicted depression score). A review of these discrepancies by 5 independently licensed clinicians, who were blinded to the administered score, yielded PHQ-8 categorization of members’ vocalizations that were consistent with the ML model categorization twice as often as they were with the administered PHQ-8 score.

## Discussion

### Principal Results

The current evaluation, leveraging speech analysis to detect depression symptoms across different levels of severity within a large real-world clinical case management context, represents, to our knowledge, the first evaluation of its kind. Overall, the findings for the combined acoustic-semantic ML model demonstrate strong model performance across a variety of key metrics. The AUROC value of approximately 0.81, the overall CCC value of 0.54, and mean of the sensitivity and specificity at the EER of 0.73 in the Blind Set demonstrate robust clinical support for the model’s ability to accurately predict severity of depression. These results compare favorably to previous research, which has primarily relied on much smaller samples and incorporated pilot, simulation, or controlled study designs [29,30]. Whereas many prior studies have focused on application of acoustic or semantic speech analysis, this study reports on an approach that combined information from both semantic and acoustic-based models. Future development and testing of speech analysis models should continue to explore the benefits of combining acoustic and semantic models.

It is noteworthy that the model performed consistently across all major subgroups and PHQ-8 classification levels, with AUROC values ranging from 0.79 to 0.85 and CCC values ranging from 0.44 to 0.61. However, the model did undercharacterize individuals at the highest PHQ-8 level (ie, “severe” depression), likely due to smaller sample size in this category of depression on which this model was trained. Among the most promising findings from the subgroup analyses was the model’s strong accuracy in predicting depression severity among older adults across key metrics. This finding is particularly significant, given that older adults have the highest rates of undetected depression [34], are often less likely to recognize or self-report symptoms of depression [35], and may experience depression with fewer dysphoric symptoms and more somatic complaints, which can be misattributed to physical illness [36,37].

The model’s ability to detect depression symptoms at lower severity levels offers significant real-world potential for early identification and person-fit and right-sized interventions earlier in individuals’ clinical trajectories. In addition to its ability to classify depression presence (PHQ-8<10 vs ≥10), the model performed well across specific PHQ-8 severity levels, particularly in the minimal and mild ranges. This suggests promising applications for early, proactive, cost-effective, and lower-intensity interventions (eg, digital interventions, BH coaching, and peer or social support) that may prevent symptom progression and reduce relapse rates. Notably, beyond the personal and clinical benefits, earlier interventions and prevention of depression may also have significant financial implications, potentially reducing health care costs associated with advanced-stage depression treatment.

Beyond BH contexts, the model’s performance in general medical (non-BH) case management calls suggests even greater potential for broadening depression detection. The model achieved a comparable level of performance (AUROC cutoff 10=0.81; range=0.79-0.85) in non-BH case manager calls, indicating potential for integrating depression detection into clinical decision-making in settings where the PHQ-9 is not routinely administered and where depression is often undetected [38]. Furthermore, the potential financial impact of enhanced depression detection in non-BH contexts is considerable, especially given that the total cost of care for members with both a BH condition and a chronic physical health condition, experienced by many members in medical-surgical case management, is approximately 3 times higher than for those with the same condition but no BH diagnosis [39,40]. The foregoing, notwithstanding, findings related to model performance in non-BH case management should be considered preliminary in light of the smaller sample size (n=110, Blind Set sample). Accordingly, additional application of speech analysis in non-BH contexts and other physical health settings is warranted.

The findings with respect to the overreporting and underreporting on the PHQ-8 offer insights into the potential for speech-based analysis to enhance depression detection objectivity [41-43]. Specifically, speech analysis may be less susceptible to bias, whether conscious or unconscious, relative to subjective report or traditional measurement. The observed trend of likely underreporting on the PHQ-8 (ie, the administered score being much lower than the predicted score) aligns with prior research on the impact of stigma, lower BH literacy, and other factors that contribute to self-report bias and underreporting of depression [44]. On the other hand, overreporting on the PHQ-9 (ie, the administered score being much higher than the predicted score) may suggest heightened or exaggerated response tendencies, personality characteristics, or efforts to obtain help [45].

Finally, this study highlights important opportunities for speech analysis and other digital phenotyping approaches to improve administrative and clinical workflows. It is noteworthy that case managers spent nearly 20% of total call time administering the PHQ-9. From an efficiency standpoint, this is time that could be better allocated to establishing a therapeutic alliance, collaborating to identify and define BH goals, addressing ambivalence and other potential obstacles to achieving those goals, and directly addressing the member’s chief concerns. Greater efficiency also establishes opportunity for case managers to interact with more members. From a clinical process standpoint, time spent administering measures such as the PHQ-9 can be awkward and even frustrating for members and may adversely affect rapport and engagement. Furthermore, inconsistencies in PHQ-9 administration can introduce errors or variability in measurement, potentially leading to misinterpretation of symptoms. In contrast, having objective, real-time data on depression severity could provide valuable insights for clinical decision-making and for providing proactive and personalized treatment plan.

### Strengths and Limitations

This study has several key strengths, including its large sample size, evaluation of speech analysis in a real-world clinical context, use of naturalistic conversations versus analog speech tasks (such as reading-defined passages or phrases, or repeating specified sounds), integration of both semantic and acoustic properties of speech, and analysis of model performance across subgroups and depression severity levels using numerous evaluation metrics. At the same time, there are several limitations that should be considered when interpreting the current findings and guiding future research.

While the large sample size included in this real-world evaluation is unique in the field of digital phenotyping [15], the sample sizes for some of the subanalyses, including the analyses of the non-BH calls and the highest PHQ-9 severity level (“Severe”) condition, were relatively small. Given this, caution should be exercised when interpreting these findings. Moreover, data on member race and ethnicity were not available. As such, the generalizability of the current results to different ethnic, cultural, and linguistic groups cannot be definitively determined. That said, the acoustic-semantic speech model was developed and trained on a very large and diverse sample [23,46].

Furthermore, prediction of depression by the model, like with any ML model, includes a degree of error or imprecision. In the current analysis, this was equivalent to approximately 4.06 points on average (the reported MAE) on the PHQ-9, which itself has imperfect accuracy [47]. As such, predicted scores should be interpreted with this in mind. With additional data, precision is likely to further increase.

In addition, the collection of the case management recordings on a single audio channel posed a challenge for this study, necessitating the use of ASR for conversion of speech to text, diarization for speaker separation, and speaker attribution labeling. While these processes generally have low error rates, they are not entirely error-free. Diarization was performed using a leading commercial tool as manual processing of calls would have required listening to and annotating thousands of hours of recordings, a time-intensive process that is also prone to errors. Additionally, the use of automatic speech processing better reflects how an actual implementation would be performed in a real-world setting. However, diarization errors (ie, poor or missing speaker separation) were encountered, and these errors propagated through the preprocessing and annotation pipeline, affecting both automated speaker attribution and removal of PHQ-8 content.

Furthermore, data curation errors observed (eg, inclusion of voicemails and conversations with minors), inevitable in a real-world dataset of this kind, may have impacted performance (in both positive and negative directions); on balance, however, these likely did not have more than a negligible effect on the reported performance metrics. Many of these challenges and resulting errors may be attenuated in prospective implementations (vs the current retrospective-focused analysis) in the future, given that (1) current call management systems routinely record speakers on separate channels, significantly mitigating the challenges of diarization (many legacy systems do not have diarization capability but some may be specially configured to do so), and (2) formal implementation of this technology within the case manager’s workflow would limit inclusion of irrelevant (eg, voicemail messages) or inappropriate (eg, minors and different languages) calls through either call management software technology or manual exclusion by the case manager according to inclusion and exclusion criteria.

### Future Studies and Real-World Deployments

The successful deployment of AI-driven speech analysis for depression detection requires careful integration into existing health care workflows. One promising approach is its integration into telehealth platforms, where it could facilitate real-time assessment and early detection during virtual consultations. Embedding the model into electronic health records, virtual scribe technology or clinical decision support systems could further enhance its use by providing clinicians with objective, data-driven insights alongside traditional assessments. This study represents a step toward the rigorous validation of AI-based health care tools, ensuring their accuracy and reliability across diverse populations. For successful deployment, ensuring security, safety, and compliance with Health Insurance Portability and Accountability Act, General Data Protection Regulation, and other data protection regulations is essential, along with continuous monitoring of system performance on test datasets to maintain reliability and accuracy. Additionally, clinician adoption depends on ensuring that the tool is user-friendly and seamlessly integrates into existing workflows without adding unnecessary burden.

Although the data used in this study are deidentified, future studies and real-world deployments should incorporate a protocol for obtaining explicit informed consent from members, ensuring ethical transparency and alignment with established guidelines for digital health interventions. One of the primary challenges in ML-based depression detection is the mitigation of bias, as algorithmic outputs may be influenced by dataset imbalances or systemic biases. In this study, bias evaluation was conducted across key demographic subgroups, but future research should expand on bias mitigation strategies and assess ethical AI deployment frameworks to ensure equitable model performance across diverse populations.

Additionally, AI governance is a critical factor in real-world deployment, necessitating adherence to key principles such as transparency, fairness, and accountability. Transparency ensures that AI models operate in a manner that is understandable, explainable, and accessible to stakeholders, including clinicians and patients. Fairness requires that models are developed and validated in a way that minimizes bias and ensures equitable performance across diverse populations, preventing disparities in mental health assessments. Accountability involves establishing clear oversight mechanisms to monitor AI decision-making, ensuring that these technologies align with ethical standards, regulatory requirements, and best practices for patient care. Future research and implementation should prioritize these principles to foster trust and reliability in AI-driven mental health tools.

Finally, while the ML model demonstrated strong predictive performance, it is important to emphasize that this tool is intended for initial assessment and triage and not for medical diagnosis. The model is designed to support early identification and risk stratification, which should be followed by clinician evaluation and judgment. This tool is not intended to replace traditional diagnostic methods.

### Conclusions

There is an urgent need to enhance detection and measurement of depression. Implementing digital phenotyping through the use of speech as a digital biomarker of depression offers significant promise for improving and accelerating depression identification and treatment. In short, the current evaluation, involving the examination of combined acoustic and semantic speech analysis for predicting depression symptom severity across PHQ-9 classification levels in a large real-world clinical context, represents the first evaluation of its type. The results reported herein provide strong support for the application and use of a readily available and unobtrusive biomarker, namely, what and how of spoken language, for detecting and measuring depression in real-world practice at this important time. This easily accessible biomarker has significant potential for application in health care settings, ranging from “preclinical” case management contexts to patient-provider interactions. It is hoped that the current findings help to advance the development and application of novel ML technologies for automating and enhancing depression symptom measurement and for informing and advancing clinical decision-making, next-best actions, and personalized treatment recommendations. Moving analysis of speech for the detection of depression symptoms—not long ago deemed science fiction—to clinical reality presents considerable opportunities for changing the paradigm of BH care to be more efficient, personalized, proactive, and upstream-focused.

## Supplementary material

10.2196/69149 Multimedia Appendix 1 Metrics explanation and model receiver operating characteristic curve performance.

## Abbreviations

ASR: automatic speech recognition
AUROC: area under the receiver operating characteristic curve
BH: behavioral health
CCC: concordance correlation coefficient
EER: equal error rate
MAE: mean absolute error
ML: machine learning
PHQ-8: Patient Health Questionnaire—8 questions
PHQ-9: Patient Health Questionnaire—9 questions
SFTP: secure file transfer protocol
SVI: Social Vulnerability Index

## References

[1] (2021). 2021 NSDUH Annual National Report. Substance Abuse and Mental Health Services Administration.

[2] Vahratian A, Blumberg SJ, Terlizzi EP, Schiller JS Symptoms of anxiety or depressive disorder and use of mental health care among adults during the COVID-19 pandemic—United States, August 2020–February 2021. MMWR Morb Mortal Wkly Rep.

[3] Ettman CK, Abdalla SM, Cohen GH, Sampson L, Vivier PM, Galea S (2020). Prevalence of depression symptoms in US adults before and during the COVID-19 pandemic. JAMA Netw Open.

[4] Wang PS, Demler O, Kessler RC (2002). Adequacy of treatment for serious mental illness in the United States. Am J Public Health.

[5] Lovett L (2023). Highmark health’s behavioral health director: personalized care, upstream prevention will define the industry. BH Business.

[6] Fahed M, McManus K, Vahia IV, Offodile AC (2022). Digital phenotyping of behavioral symptoms as the next frontier for personalized and proactive cancer care. JCO Clin Cancer Inform.

[7] Huckvale K, Venkatesh S, Christensen H (2019). Toward clinical digital phenotyping: a timely opportunity to consider purpose, quality, and safety. NPJ Digit Med.

[8] Insel TR (2017). Digital phenotyping: technology for a new science of behavior. JAMA.

[9] Mohr DC, Shilton K, Hotopf M (2020). Digital phenotyping, behavioral sensing, or personal sensing: names and transparency in the digital age. NPJ Digit Med.

[10] Chia AZR, Zhang MWB (2022). Digital phenotyping in psychiatry: a scoping review. Technol Health Care.

[11] Herrman H, Patel V, Kieling C (2022). Time for united action on depression: a Lancet–World Psychiatric Association Commission. Lancet.

[12] Galatzer-Levy IR, Onnela JP (2023). Machine learning and the digital measurement of psychological health. Annu Rev Clin Psychol.

[13] Low DM, Bentley KH, Ghosh SS (2020). Automated assessment of psychiatric disorders using speech: a systematic review. Laryngoscope Investig Otolaryngol.

[14] Koutsouleris N, Hauser TU, Skvortsova V, De Choudhury M (2022). From promise to practice: towards the realisation of AI-informed mental health care. Lancet Digit Health.

[15] Flanagan O, Chan A, Roop P, Sundram F (2021). Using acoustic speech patterns from smartphones to investigate mood disorders: scoping review. JMIR Mhealth Uhealth.

[16] Shen Y, Yang H, Lin L Automatic depression detection: an emotional audio-textual corpus and a GRU/bilstm-based model.

[17] Cohen AS, Cox CR, Le TP (2020). Using machine learning of computerized vocal expression to measure blunted vocal affect and alogia. NPJ Schizophr.

[18] El-Den S, Chen TF, Gan YL, Wong E, O’Reilly CL (2018). The psychometric properties of depression screening tools in primary healthcare settings: a systematic review. J Affect Disord.

[19] Kroenke K, Spitzer RL, Williams JB (2001). The PHQ-9: validity of a brief depression severity measure. J Gen Intern Med.

[20] Kroenke K, Spitzer RL (2002). The PHQ-9: a new depression diagnostic and severity measure. Psychiatr Ann.

[21] Rutowski T, Shriberg E, Harati A, Lu Y, Oliveira R, Chlebek P Cross-demographic portability of deep NLP-based depression models.

[22] Harati A, Shriberg E, Rutowski T, Chlebek P, Lu Y, Oliveira R (2021). Speech-based depression prediction using encoder-weight-only transfer learning and a large corpus.

[23] Lin D, Nazreen T, Rutowski T (2022). Feasibility of a machine learning-based smartphone application in detecting depression and anxiety in a generally senior population. Front Psychol.

[24] Baevski A, Zhou Y, Mohamed A, Auli M (2020). Wav2vec 2.0: a framework for self-supervised learning of speech representations. Adv Neural Inf Process Syst.

[25] Beltagy I, Peters ME, Cohan A (2020). Longformer: the long-document transformer. arXiv.

[26] (2024). Speech to text—Amazon Transcribe—AWS. Amazon Web Services, Inc.

[27] (2018). Introducing medical language processing with Amazon Comprehend Medical | AWS ML blog. Amazon Web Services, Inc.

[28] Fara S, Goria S, Molimpakis E, Cummins N (2022). Interspeech 2022.

[29] Seneviratne N, Espy-Wilson C (2022). Multimodal depression severity score prediction using articulatory coordination features and hierarchical attention based text embeddings.

[30] Wang J, Ravi V, Flint J, Alwan A (2022). Unsupervised instance discriminative learning for depression detection from speech signals.

[31] (2024). Social Vulnerability Index (CDC/ATSDR SVI). CDC/ATSDR.

[32] Bn S, Abdullah S (2022). Privacy sensitive speech analysis using federated learning to assess depression.

[33] Rutowski T, Harati A, Shriberg E, Lu Y, Chlebek P, Oliveira R (2022). Toward corpus size requirements for training and evaluating depression risk models using spoken language.

[34] Zenebe Y, Akele B, W/Selassie M, Necho M (2021). Prevalence and determinants of depression among old age: a systematic review and meta-analysis. Ann Gen Psychiatry.

[35] Devita M, De Salvo R, Ravelli A (2022). Recognizing depression in the elderly: practical guidance and challenges for clinical management. Neuropsychiatr Dis Treat.

[36] Gottfries CG (1998). Is there a difference between elderly and younger patients with regard to the symptomatology and aetiology of depression?. Int Clin Psychopharmacol.

[37] Hegeman JM, Kok RM, van der Mast RC, Giltay EJ (2012). Phenomenology of depression in older compared with younger adults: meta-analysis. Br J Psychiatry.

[38] Ducat L, Philipson LH, Anderson BJ (2014). The mental health comorbidities of diabetes. JAMA.

[39] Davenport S, Gray T, Melek S, Milliman Research Report (2020). Milliman high-cost patient study 2020. https://www.milliman.com/-/media/milliman/pdfs/articles/milliman-high-cost-patient-study-2020.pdf.

[40] Bellon J, Quinlan C, Taylor B, Nemecek D, Borden E, Needs P (2022). Association of outpatient behavioral health treatment with medical and pharmacy costs in the first 27 months following a new behavioral health diagnosis in the US. JAMA Netw Open.

[41] Malpass A, Dowrick C, Gilbody S (2016). Usefulness of PHQ-9 in primary care to determine meaningful symptoms of low mood: a qualitative study. Br J Gen Pract.

[42] Thombs BD, Kwakkenbos L, Levis AW, Benedetti A (2018). Addressing overestimation of the prevalence of depression based on self-report screening questionnaires. CMAJ.

[43] De Jong MG, Fox JP, Steenkamp J (2015). Quantifying under- and overreporting in surveys through a dual-questioning-technique design. J Mark Res.

[44] Hunt J, Eisenberg D (2010). Mental health problems and help-seeking behavior among college students. J Adolesc Health.

[45] Ma S, Kang L, Guo X (2021). Discrepancies between self-rated depression and observed depression severity: the effects of personality and dysfunctional attitudes. Gen Hosp Psychiatry.

[46] Harati A, Rutowski T, Lu Y, Obeid I, Picone J, Selesnick I (2022). Biomedical Sensing and Analysis.

[47] Levis B, Benedetti A, Thombs BD (2019). Accuracy of Patient Health Questionnaire-9 (PHQ-9) for screening to detect major depression: individual participant data meta-analysis. BMJ.

