# Supplementary material for: Digital Phenotyping for Detecting Depression Severity in a Large Payor-Provider System: Retrospective Study of Speech and Language Model Performance
Source: JMIR AI. 2025 Jun 19;4:e69149. doi: 10.2196/69149 (PMC12223686; doi:10.2196/69149)
Supplement: Multimedia Appendix 1 [file ai-v4-e69149-s001.docx]

**Metrics Explanation and Model ROC Performance**

​The ML model results were calculated and included CCC, mean absolute error (MAE), area under the receiver operating curve (ROC-AUC), and sensitivity and specificity at the point of equal error (EER) for the Dev Set and Blind Set. The point of Equal Error is the location along the Receiver Operating Characteristic curve at which the false positive rate is equal to the false negative rate. The regression metrics, CCC and MAE, were calculated for this study to show performance of the ML model. The CCC measures the correlation between a gold standard (in this case the PHQ-8 score) and the model prediction and has a range from -1 to 1, where 1 denotes complete positive correlation and 0 is no better than chance [1]. The MAE regression metric is the mean difference between the actual values (in this case the PHQ8 score) and the predicted values (in this case the model prediction). MAE can be subject to bias based on the sample distribution of depression severity scores and is lower (i.e., better) when samples have fewer severe cases given that the ML model evaluated in the present study was developed on samples with fewer severe cases. MAE was calculated for the Highmark Health data, which had relatively equal distributions of subjects in each severity category (i.e., more severe cases than the development samples), potentially inflating MAE in the present study.  All classification analyses were conducted with the PHQ-8 as the criterion or observed score. Predicted scores were derived directly from the ML models and then binned according to the following PHQ-8 depression severity classifications: none or minimal (0 - 4), mild (5 - 9), moderate (10 - 14), moderately severe (15 - 19), and severe (20 - 24), producing a five-way classification. Next, ROC analyses were conducted, comparing predicted to observed scores across five binary classifications at the four PHQ-8 cut-offs (5, 10, 15, 20): 0 to 4 versus 5 to 24, 0 to 9 versus 10 to 24, 0 to 14 versus 15 to 24, 0 to 19 versus 20 to 24. ROC-AUC and sensitivity and specificity at the rate of equal error were calculated at each cut-off and reported for the ML model.  Figures S1-S12 illustrate comparisons of relevant ROC curves (overall and per subgroup on Blind Set).

​

​

**Figure S1**. ROC for Blind Set, age ≤ 40 years


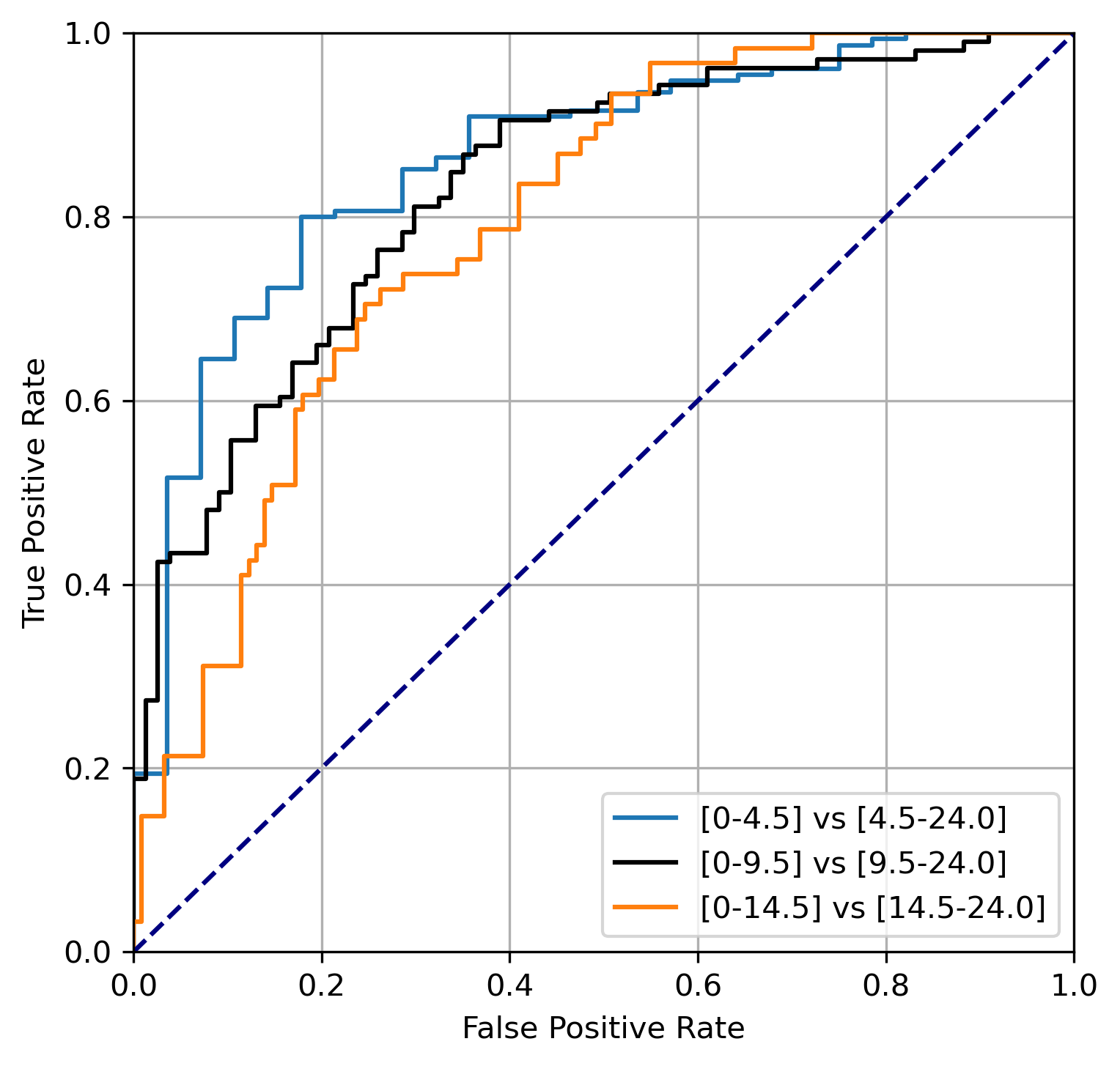


**​**

**Figure S2.** ROC for Blind Set, age 40-64 years

​

​
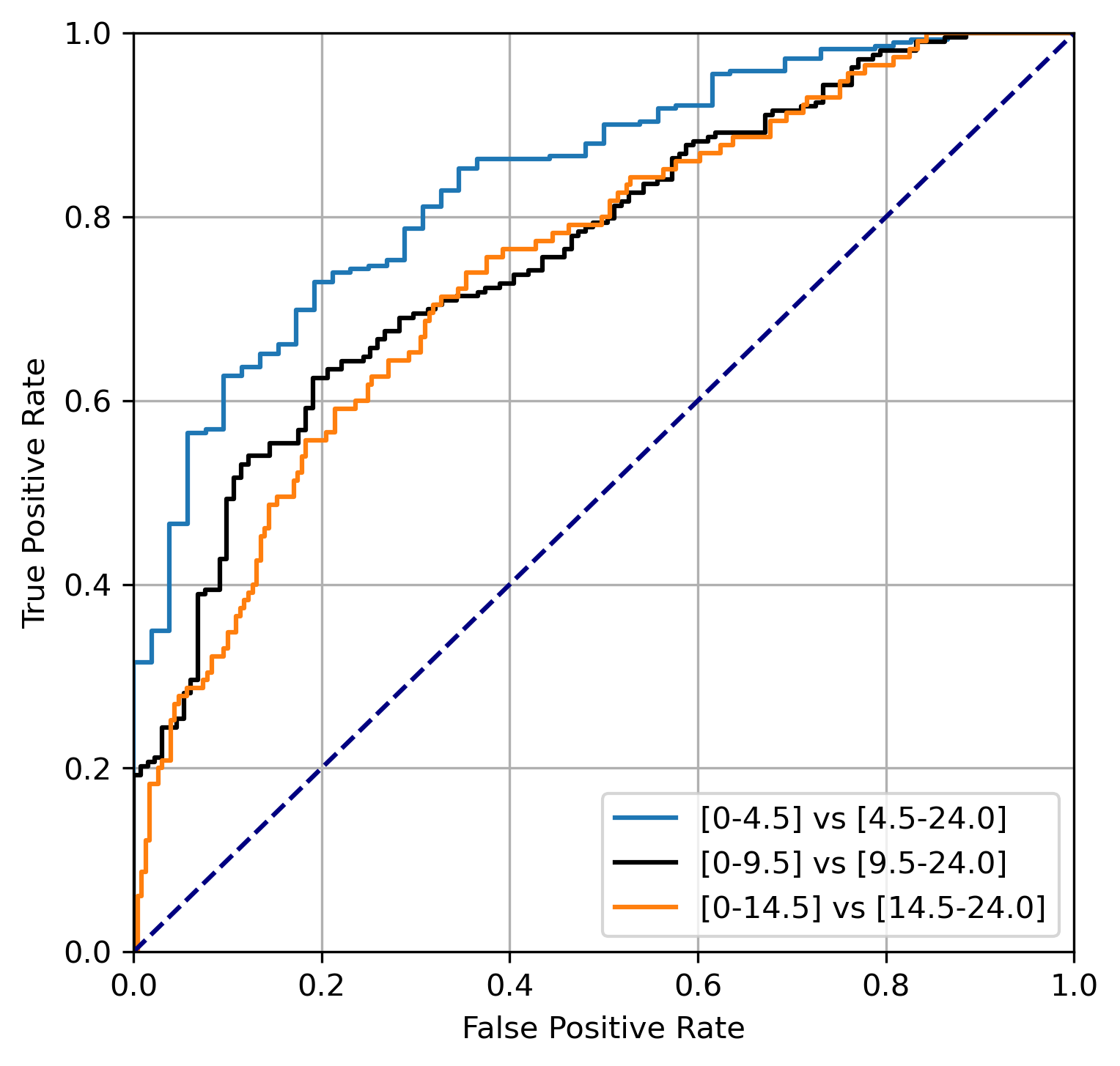


​

**​**

**Figure S3.** ROC for Blind Set, age 65+ years

​
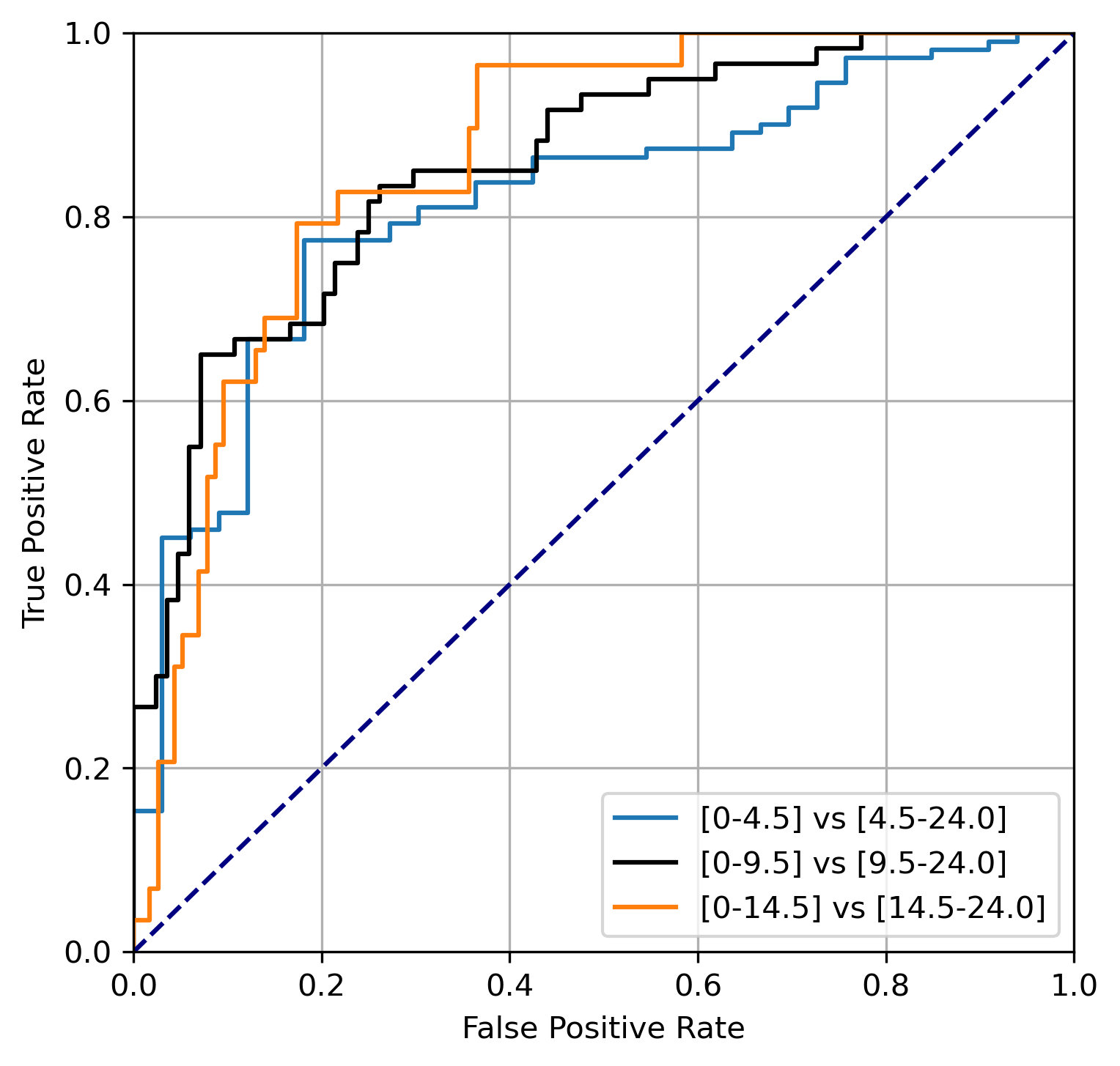


​

**​**

**Figure S4.** ROC for Blind Set, male sex

​
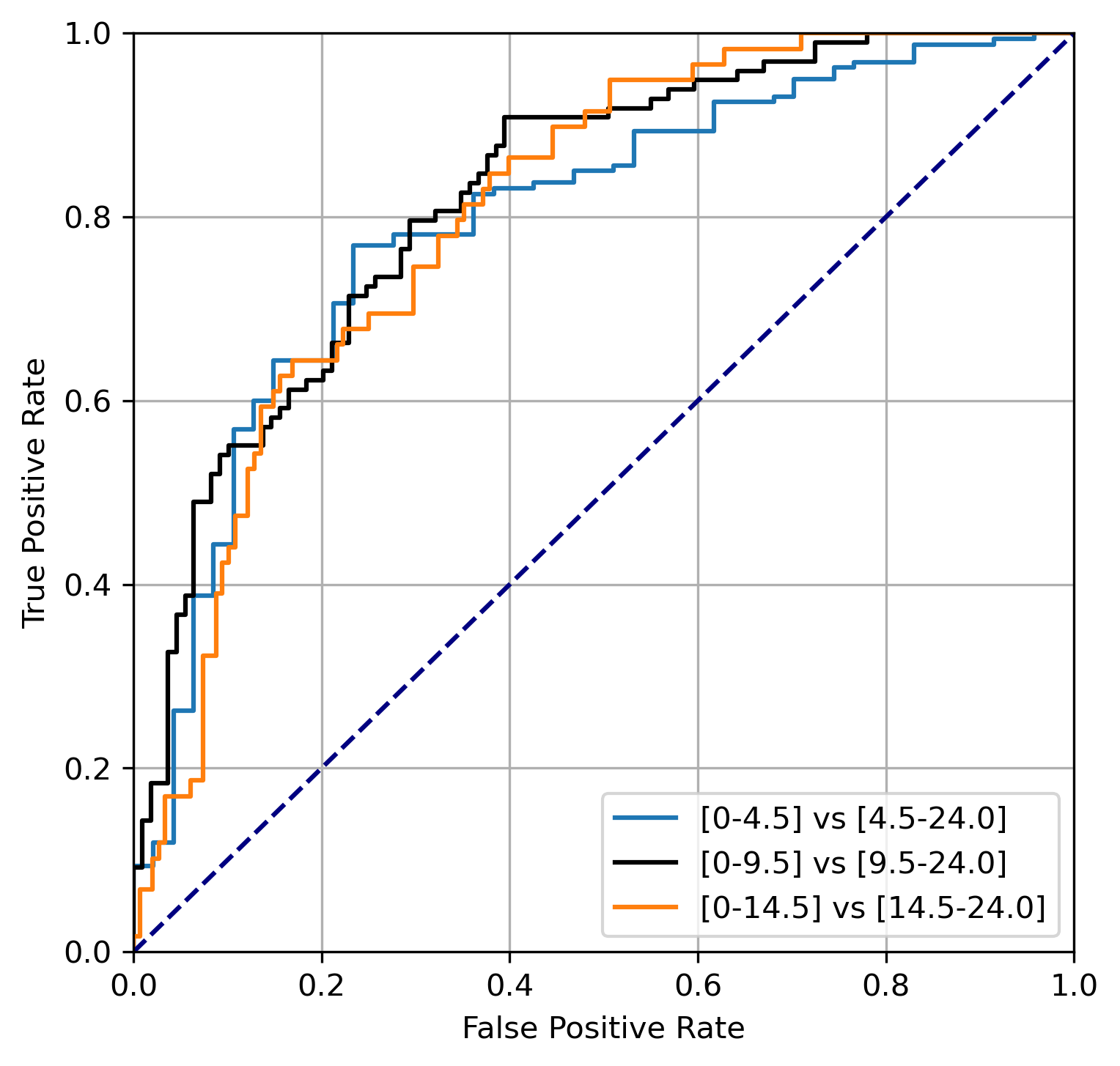


**​**

**Figure S5.** ROC for Blind Set, female sex

​
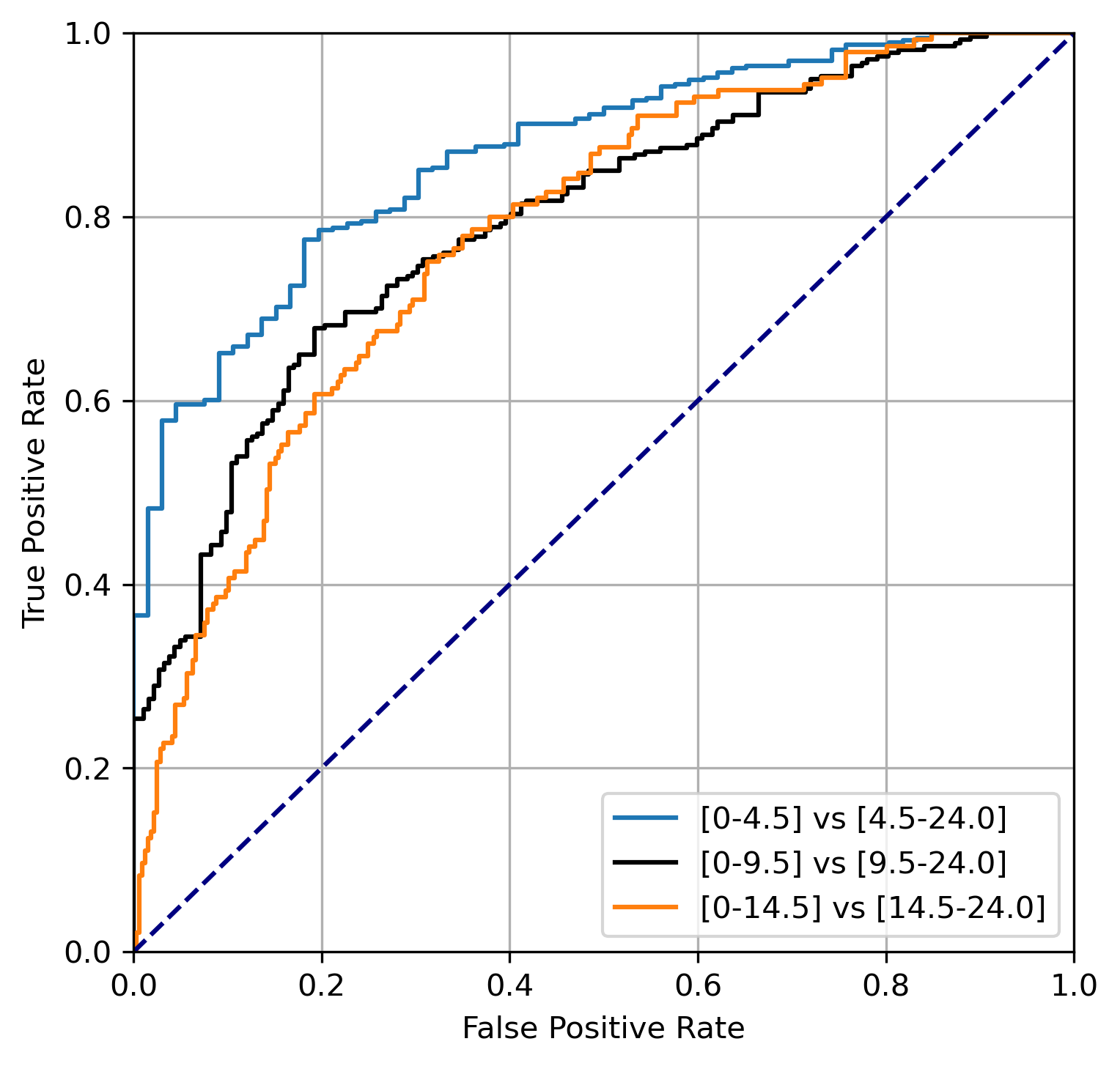


​

**​**

**Figure S6.** ROC for Blind Set, BH CM calls

​
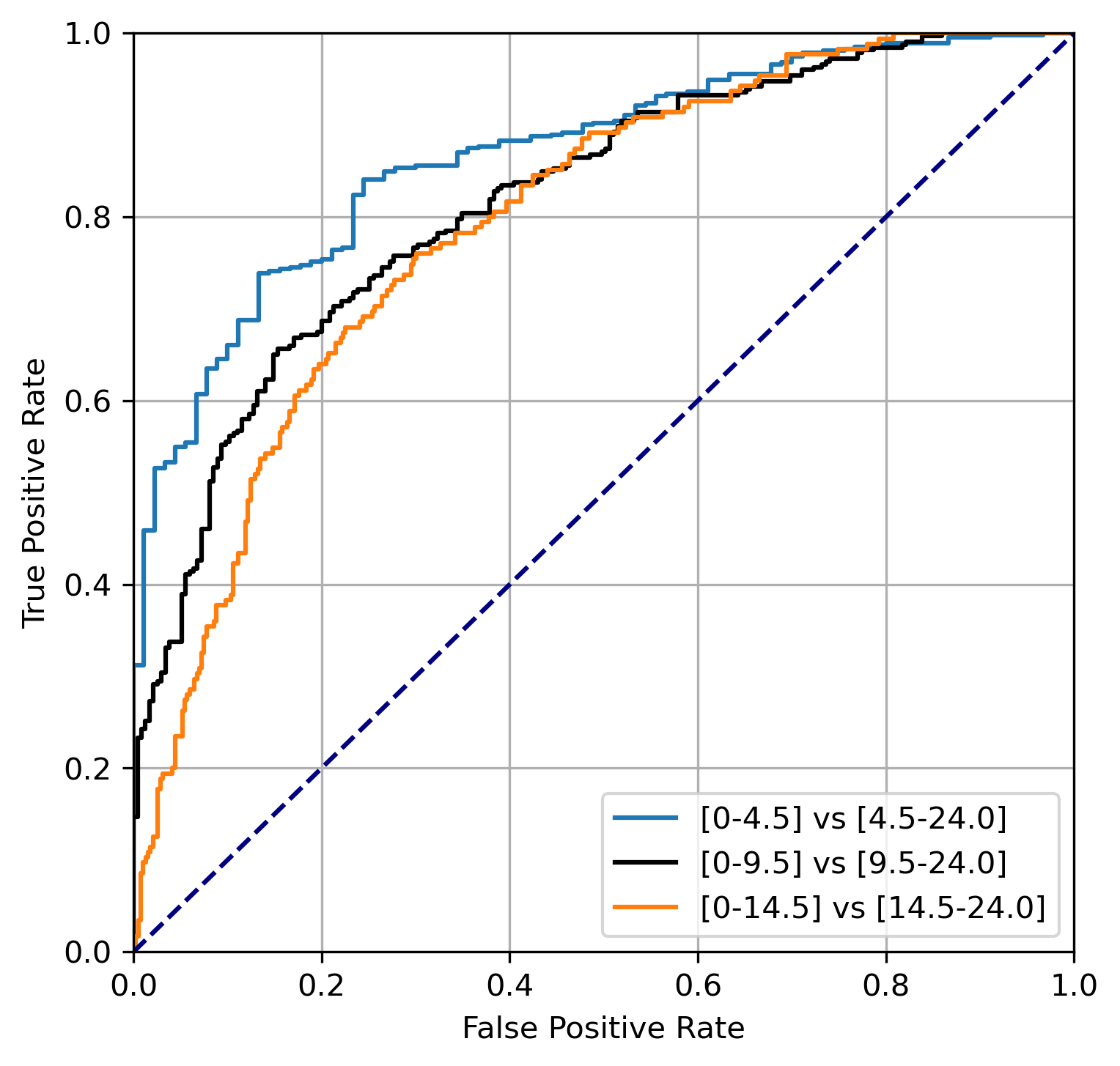


**​**

**Figure S7.** ROC for Blind Set, non-BH CM calls

​
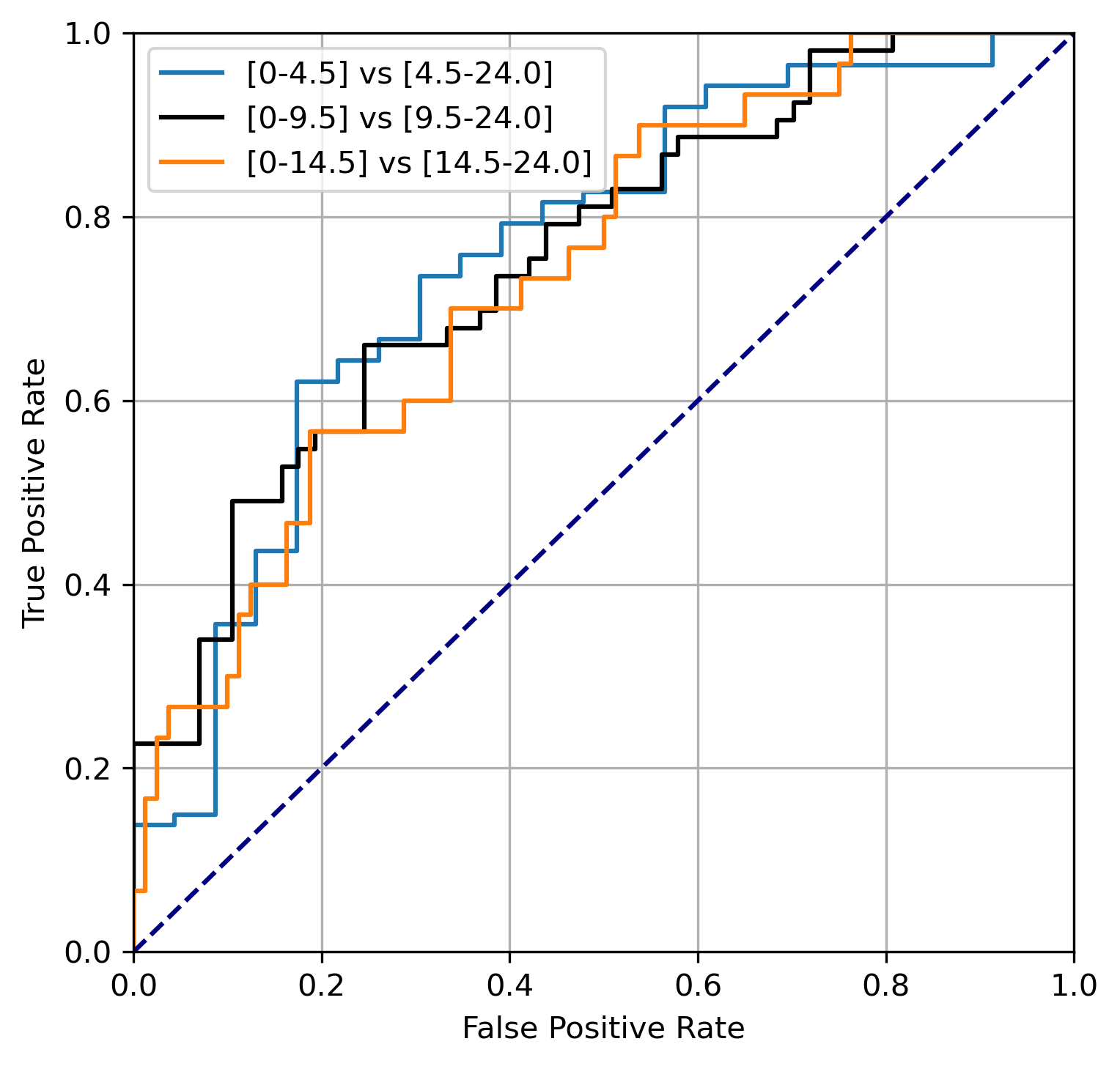


​

**​**

**Figure S8.** ROC for Blind Set, SVI=1

​
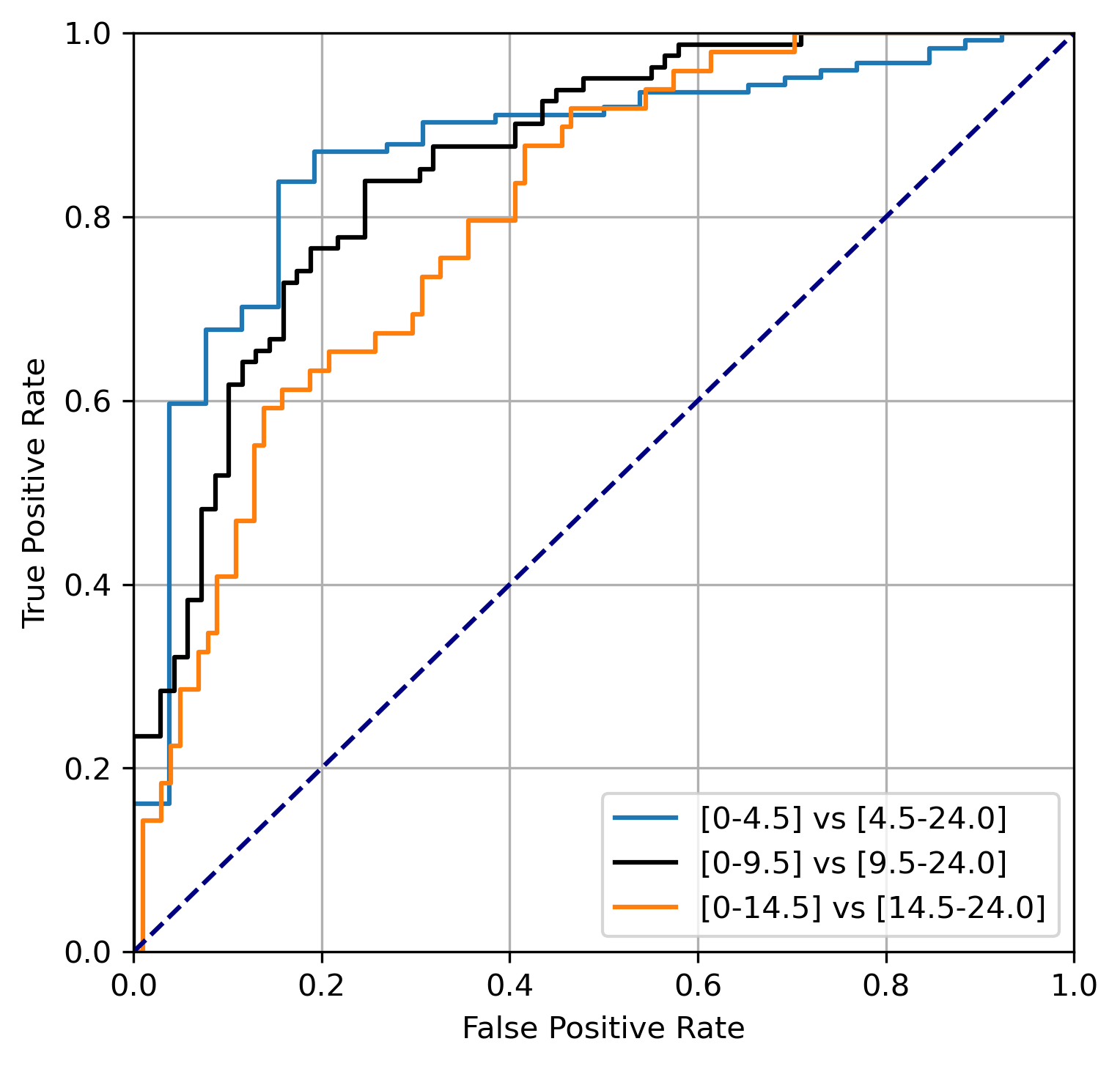


**​**

**Figure S9.** ROC for Blind Set, SVI=2

​
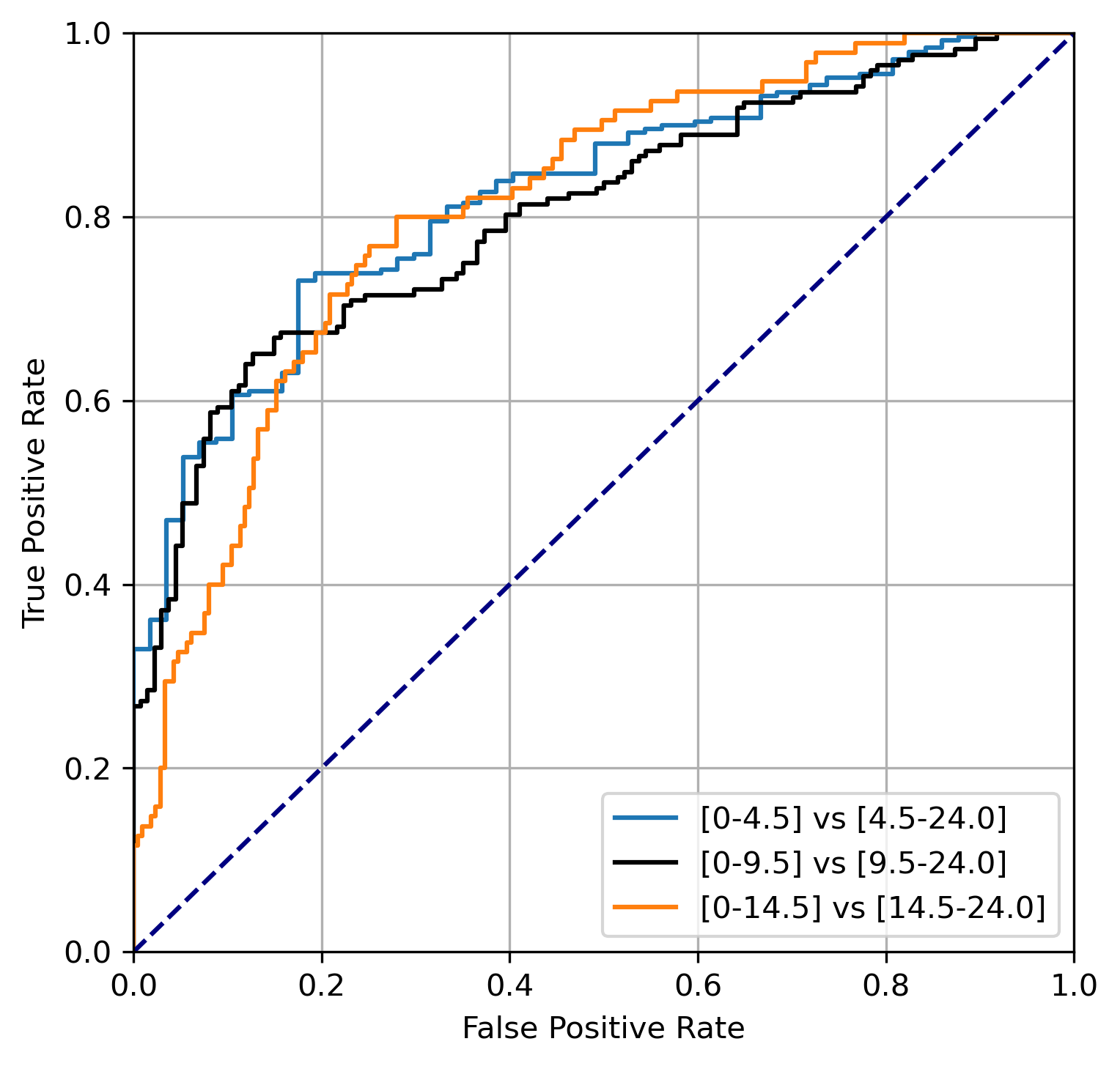


​

**​**

**Figure S10.** ROC for Blind Set, SVI=3


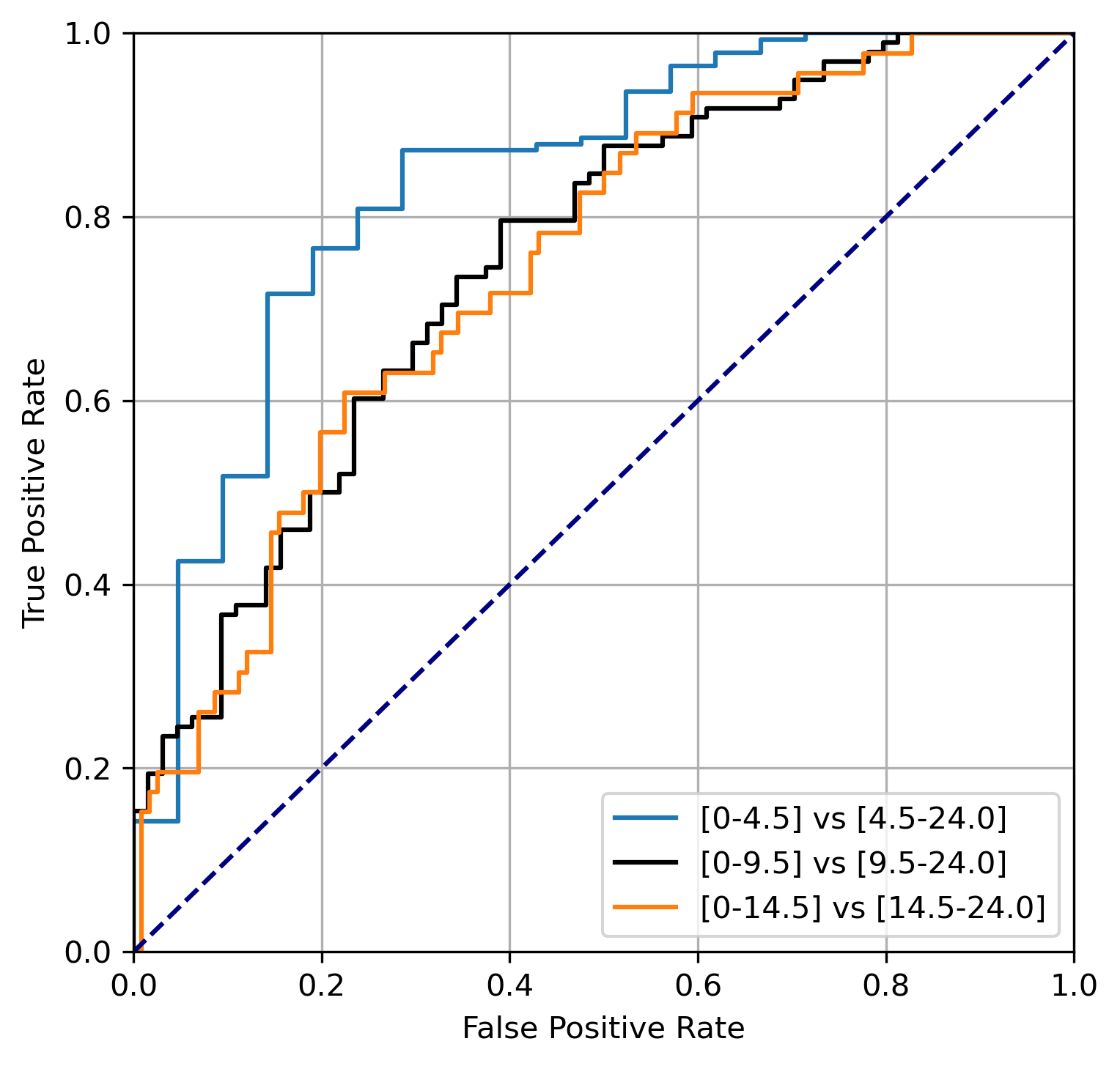


**​**

**Figure S11.** ROC for Blind Set, SVI=4

​
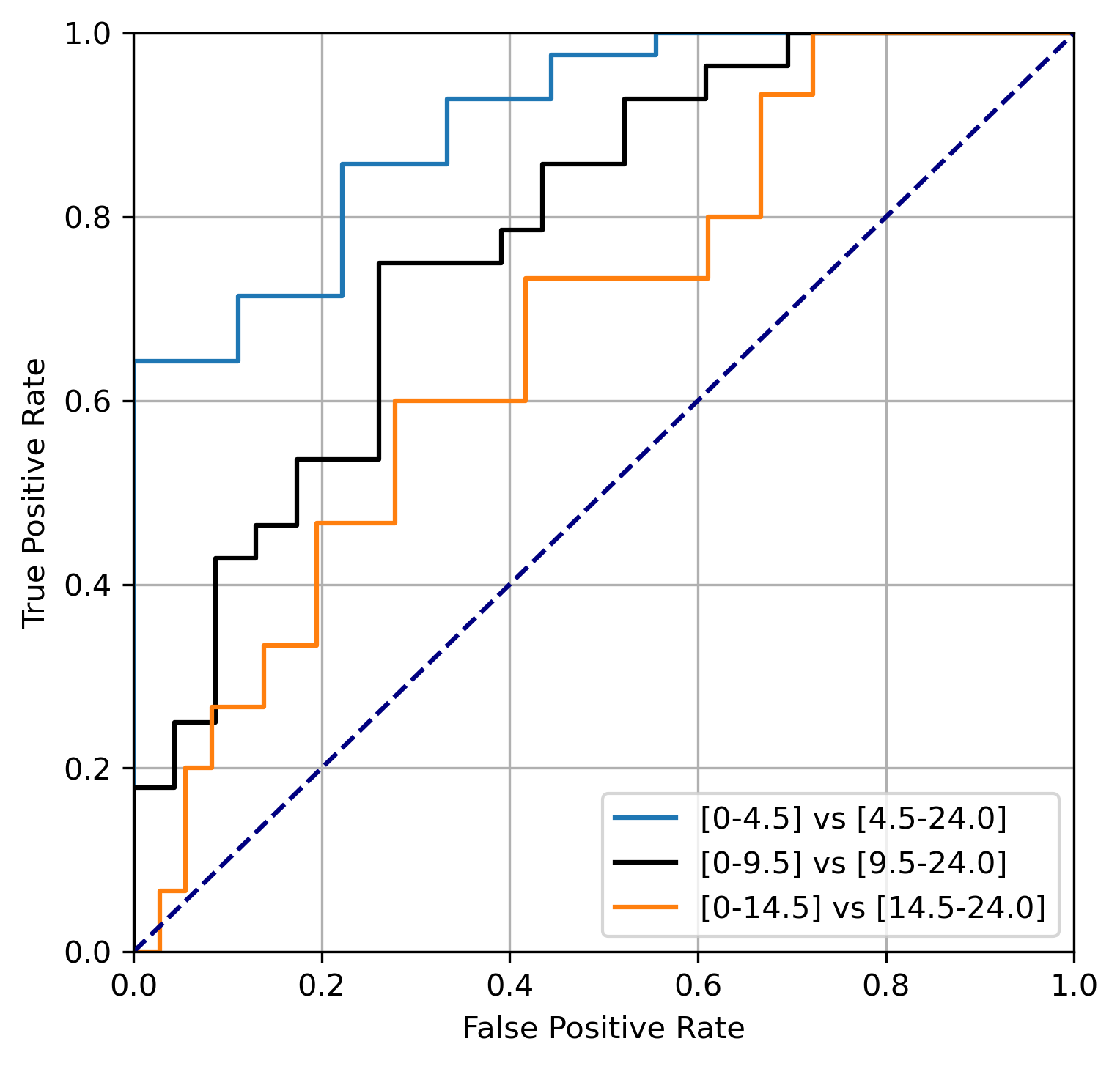


​

**​**

**Figure S12.** ROC for overall Blind Set

​
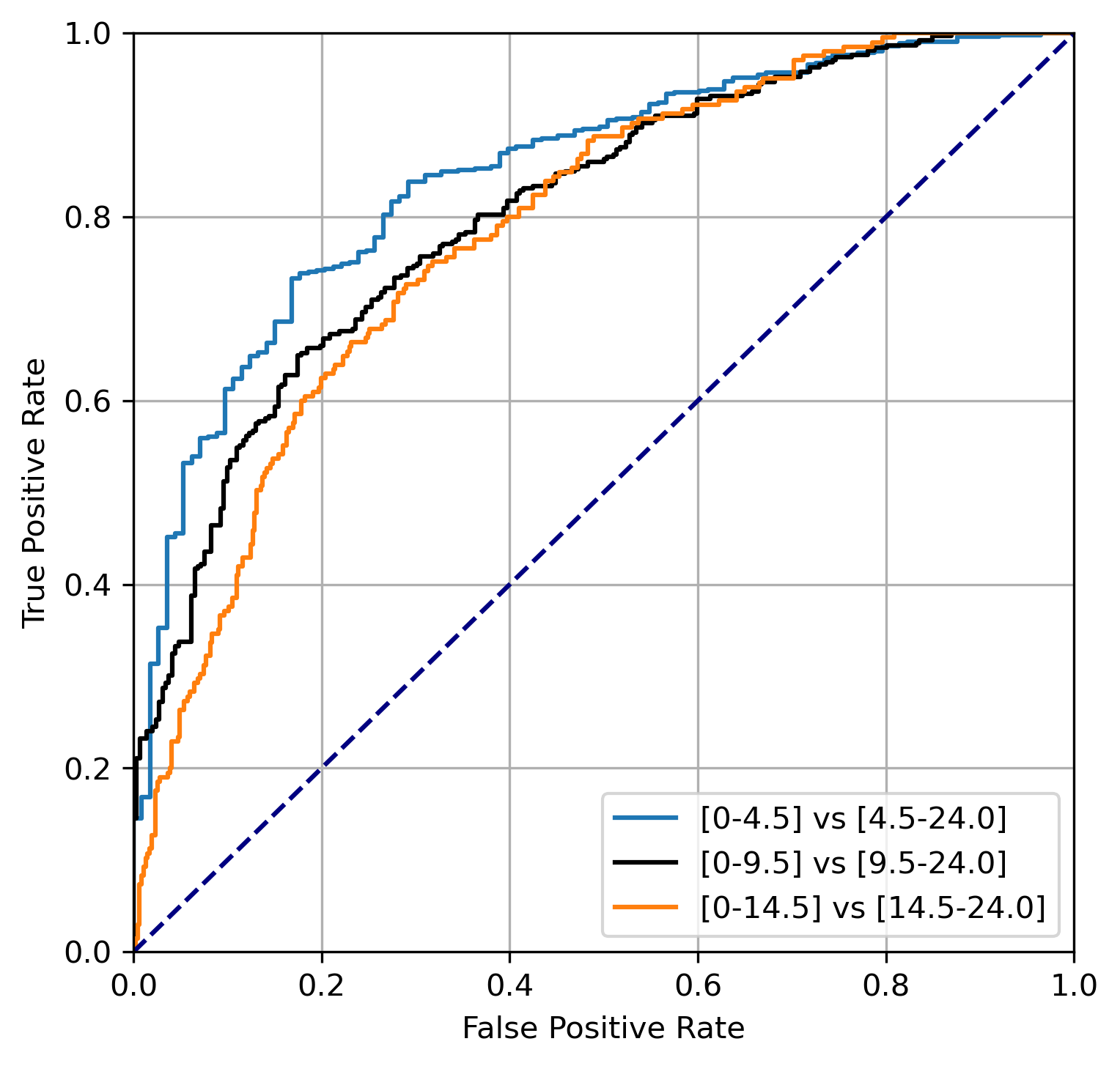


​

**​References**

​

1. ​Lin LIK. A concordance correlation coefficient to evaluate reproducibility. Biometrics. 1989;45:255. doi:10.2307/2532051

**​**
